# A MODEL OF CARE FOR OLDER ADULTS WITH IMPAIRED VISION OR BLINDNESS AT THE END OF LIFE

**DOI:** 10.1093/geroni/igz038.2031

**Published:** 2019-11-08

**Authors:** Ximena Morales, Ricardo Blondet, Marcos Milanez, Deepak Mandi, Gabino Lares, Michael A Silverman

**Affiliations:** 1 Trustbridge Hospice, West Palm Beach, Florida, United States; 2 West Palm Beach VA Medical Center, West Palm Beach, Florida, United States

## Abstract

Our objectives were to describe unique challenges of visually-impaired patients receiving end-of-life institutional care and to propose a model for the comprehensive care of the vision-impaired patients incorporating bedside techniques and advanced assistive technology. The prevalence of visual impairment in long-term care is increasing. Collaborating with our Blind Rehabilitation Center, we have summarized a care model including the identification of patients who have impaired vision and adjustments of daily routine. Care was consistently provided by staff with voices familiar to patients. Staff is trained to introduced themselves clearly by voice when entering the room. Patients engage in hobbies that are less dependent on vision, such as music therapy. Safety measures are taken to facilitate mobility. We describe the case of a 90-year-old WWII Veteran with dementia and dysphagia who was legally blind and required extensive assistance with his ADLs. Although initially calm, the patient eventually became delirious, reliving his time as a gunner in the Navy and believing he was firing on Japanese Kamikaze planes. After his visual impairment was addressed using the approach described above, the patient became calmer. Listening to his wife’s voice and enjoying his favorite gospel music helped him cope better with the situation until he died peacefully on hospice care. In conclusion, a model of care considering visual impairment was effective at alleviating distress. More emphasis needs to be placed on evaluating and managing sensory impairment when providing care for older adults approaching the end of life.

